# Household composition and anxiety symptoms during the COVID-19 pandemic: A population-based study

**DOI:** 10.1371/journal.pone.0277243

**Published:** 2022-11-03

**Authors:** André J. McDonald, Hayley A. Hamilton, Tara Elton-Marshall, Yeshambel T. Nigatu, Damian Jankowicz, Susan J. Bondy, Samantha Wells, Christine M. Wickens

**Affiliations:** 1 Institute for Mental Health Policy Research, Centre for Addiction and Mental Health, Toronto, Ontario, Canada; 2 Dalla Lana School of Public Health, University of Toronto, Toronto, Ontario, Canada; 3 Campbell Family Mental Health Research Institute, Centre for Addiction and Mental Health, Toronto, Ontario, Canada; 4 Department of Epidemiology and Biostatistics, Schulich School of Medicine and Dentistry, Western University, London, Ontario, Canada; 5 Faculty of Medicine, School of Epidemiology and Public Health, University of Ottawa, Ottawa, Ontario, Canada; 6 Department of Health Sciences, Lakehead University, Thunder Bay, Ontario, Canada; 7 Information Management, Centre for Addiction and Mental Health, Toronto, Ontario, Canada; 8 Department of Psychiatry, University of Toronto, Toronto, Ontario, Canada; 9 School of Psychology, Deakin University, Burwood, Victoria, Australia; 10 Institute of Health Policy, Management and Evaluation, University of Toronto, Toronto, Ontario, Canada; 11 Department of Pharmacology and Toxicology, University of Toronto, Toronto, Ontario, Canada; PGIMER: Post Graduate Institute of Medical Education and Research, INDIA

## Abstract

**Introduction:**

Household composition may be an important factor associated with anxiety during the COVID-19 pandemic as people spend more time at home due to physical distancing and lockdown restrictions. Adults living with children–especially women–may be particularly vulnerable to anxiety as they balance additional childcare responsibilities and homeschooling with work. The objective of this study was to examine the association between household composition and anxiety symptoms during the COVID-19 pandemic and explore gender as an effect modifier.

**Methods:**

Data were derived from seven waves of a national online survey of Canadian adults aged 18+ years from May 2020 to March 2021, which used quota sampling by age, gender, and region proportional to the English-speaking Canadian population (n = 7,021). Multivariable logistic and modified least-squares regression models were used.

**Results:**

Compared to those living alone, significantly greater odds of anxiety symptoms were observed among single parents/guardians (aOR = 2.00; 95%CI: 1.41–2.84), those living with adult(s) and child(ren) (aOR = 1.39; 95%CI: 1.10–1.76), and those living with adult(s) only (aOR = 1.22; 95%CI: 1.00–1.49). Gender was a significant effect modifier on the additive scale (p = 0.0487) such that the association between living with child(ren) and anxiety symptoms was stronger among men than women.

**Conclusion:**

Additional tailored supports are needed to address anxiety among adults living with children–especially men–during the COVID-19 pandemic and future infectious disease events.

## Introduction

The COVID-19 pandemic has caused many to experience social isolation due to physical distancing and lockdown restrictions, financial worry due to lost jobs and economic uncertainty, fear of becoming ill or dying from COVID-19, and grief for lost loved ones [1]. These pandemic-related stressors, among others, have contributed to increased anxiety at the population level [2]. Consequently, a large segment of the population may be at greater risk of adverse health outcomes associated with heightened anxiety both in the short term (e.g., suicide, substance misuse, overdose, cardiovascular disease, etc.) and the long term (e.g., development of chronic mental health problems) [3–5].

Parents (and other adults) living with children may be particularly vulnerable to heightened anxiety during the pandemic because of additional sources of stress affecting their lives [6, 7]. With intermittent closures of schools, daycare centres, and youth programs, parents have had to take on additional responsibilities in the home. For much of the COVID-19 pandemic, physical distancing restrictions have prevented children from socializing with their peers in person, placing pressure on parents to find ways to keep their children entertained throughout the day. Many parents have had to balance work responsibilities–often from home–with schooling their children. For single parents, balancing these additional responsibilities can be especially challenging given that they are unable to share these activities with another adult in the home. Meanwhile, parents who have lost their jobs or were already unemployed before the pandemic may be worried about their ability to provide financially for their children.

Given the unique stressors that parents living with children are experiencing and the increased time that people are spending at home together during the COVID-19 pandemic [8], household composition (i.e., living alone, living with children, or living with other adults) may be an important factor associated with anxiety. Pre-pandemic studies suggest that parents living with children do not have a greater likelihood of experiencing anxiety compared to non-parents in the general population [9, 10], while single parents and individuals living alone have been found to be at elevated risk of anxiety, making them vulnerable subpopulations of interest [9–13]. However, previous research is perhaps not generalizable to people’s experiences during the COVID-19 pandemic, which has had a substantial impact on stress in many households. Moreover, the COVID-19 pandemic has exacerbated gender disparities, which may be contributing to gender differences in parent mental health [14]. In general, women have been more likely to report depression, anxiety, loneliness, and other mental health problems compared to men during the pandemic [15–17]. In addition, mothers living with children, who prior to the pandemic already shouldered the majority of childcare responsibilities, have taken on the bulk of pandemic-related childcare responsibilities (e.g., homeschooling), likely due to traditional gender roles [14, 18]. However, some research suggests that, even though fathers are taking on less pandemic-related childcare responsibilities than mothers, the additional burden may be more detrimental to fathers’ mental health compared to that of mothers [14]. Gender could therefore moderate the relationship between household composition and anxiety. The objective of this study was to examine whether household composition was associated with anxiety symptoms during the COVID-19 pandemic in the general population and explore gender as an effect modifier.

## Methods

### Sample

This cross-sectional study is based on seven waves of a national online survey series among Canadian adults aged 18 years and older conducted by the Centre for Addiction and Mental Health in collaboration with Methodify by Delvinia [19]. The first six waves were conducted in 2020 from May 8 to May 12 (Wave 1), May 28 to June 1 (Wave 2), June 19 to 23 (Wave 3), July 10 to 14 (Wave 4), September 18 to 22 (Wave 5), and November 27 to December 1, 2020 (Wave 6). Wave 7 was conducted from March 19 to 23, 2021. Invitations to participate in the survey were sent via email to participants in the AskingCanadians online research panel that includes over 1 million Canadian members from across the country. Members of the AskingCanadians panel were initially recruited through loyalty partnerships with major Canadian corporations such as department stores, airlines, and retailers. All respondents provided electronically written informed consent at the beginning of the online survey and received loyalty points for their time and participation in the study. The Centre for Addiction and Mental Health’s Research Ethics Board approved survey data collection. Respondents were invited based on quota sampling by age, gender, and region proportional to the English-speaking Canadian population. Participants could only respond to the survey once. The overall response rate for the seven waves was 16.1%. In total, the seven waves had 1005, 1002, 1005, 1003, 1003, 1003, and 1000 respondents respectively, contributing to a pooled sample size of 7,021. Participants with missing data were excluded from regression analyses reducing the sample size to 6,739.

### Measures

Anxiety symptoms were measured with the Generalized Anxiety Disorder-7 (GAD-7) questionnaire [20]. The GAD-7 is a well-validated screening tool designed to detect anxiety symptoms based on criteria from the Diagnostic and Statistical Manual of Mental Disorders, 4^th^ Edition (DSM-IV) [21, 22]. It includes 7 items, each beginning with: “Over the last 2 weeks, how often have you been bothered by the following problems?” Response categories are on a 4-point scale ranging from (0) “Not at all” to (3) “Nearly every day”. The established cut-off score of 10 or greater was used to identify individuals with moderate to severe anxiety symptoms [20]. This cut-off score has been validated in adults, with a sensitivity of 89% and specificity of 82% [20].

Household composition was based on the combination of two questions regarding the total number of household members and total number of children under 18 years of age in the household. By subtracting the total number of children from the total number of household members, we created a categorical variable composed of four categories–live alone, live with adult(s) only, single parent/guardian, and live with adult(s) and child(ren).

Sociodemographic variables included age (18 to 29 years; 30 to 39 years; 40 to 49 years; 50 to 59 years; 60 to 69 years, and 70 years and over), gender (male, female, non-binary), marital status (married or living with partner, single/divorced/separated/widowed), educational attainment (high school or less, some post-secondary, college degree/diploma, university degree/diploma), household income in Canadian dollars (less than $40,000; $40,000-$79,999; $80,000-$119,999; $120,000+; prefer not to answer), and rurality (urban, suburban, rural).

### Analysis

Pearson’s chi-squared test was used to assess for independence of covariates. Unadjusted logistic regression models were used to estimate crude odds ratios for all covariates and anxiety symptoms. Multivariable logistic regression was then used to estimate the association between household composition and moderate to severe anxiety symptoms, controlling for age, gender, educational attainment, household income, rurality, marital status, and survey wave. We included demographic (age, gender, marital status, and rurality) and socioeconomic status (educational attainment and household income) measures in the multivariable model based on previous literature [10, 13]. We subsequently collapsed “single parents/guardians” and “live with adult(s) and child(ren)” into one category (“live with child(ren)”) and re-ran the regression model to compare “live with child(ren)” and “live with adult(s) only.” For both models, multicollinearity was assessed by examining variance inflation factors.

Given the potential importance of traditional gender roles for childcare responsibilities during the pandemic, we also tested whether gender was an effect modifier for the association between household composition and anxiety symptoms. We assessed effect modification on the additive scale by directly estimating prevalence differences. Effect modification on the additive scale is more relevant to public health because it allows for the identification of subgroups that would benefit most from intervention [23]. We first ran a binomial identity model, and then a Poisson identity model [24], which both failed to converge. We subsequently ran a modified least-squares regression model with a Normal distribution error and identity link that successfully converged. Linear regression with robust standard errors, also known as modified least-squares regression, has been shown to accurately estimate prevalence differences with binary outcome data [25]. This method corrects for the misspecified error term using the Huber-White sandwich estimator, which produces appropriate coverage of confidence intervals [24, 25]. The non-binary gender group was excluded from interaction analyses due to small sample size (n = 55). Because of the small number of single parents/guardians, we used the collapsed household composition variable described above to increase the power of the interaction analysis.

A graph was used to facilitate interpretation of interaction terms and express the association between household composition and anxiety symptoms conditional on gender. We used multivariable logistic regression to estimate adjusted probabilities of anxiety symptoms for all levels of the interaction with covariates set to their reference levels (i.e., 70+ years old, married/living with partner, university degree, $120k+ income, rural area, and survey wave 1). Unlike the modified least-squares model that tested for additive interaction using a 3-category household composition variable, we used the original 4-category household composition variable for the graph to elucidate differences between single parents/guardians and those living with adult(s) and child(ren) among men and women. All statistical analyses were conducted using SAS software, Version 9.4 [26].

## Results

The characteristics of the study sample are summarized in Table 1. Overall, 21.7% of respondents met criteria for moderate to severe anxiety symptoms. Chi-square tests showed that household composition, age, gender, marital status, household income, rurality, and survey wave were all significantly related to anxiety symptoms.

**Table 1:** Sample characteristics by anxiety symptoms, Canadian adults aged 18+ years during the COVID-19 pandemic.

Variables	Total sample	Moderate to severe anxiety symptoms (GAD-7 10+)		No to mild anxiety symptoms (GAD-7 <10)		*χ*^2^ p-value
	n = 7,021	n = 1,525		n = 5,496		
	n	n	(%)	n	(%)	
**Household composition**						<0.001
Live alone	1450	292	(20.1%)	1158	(79.9%)	
Single parent/guardian	170	66	(38.8%)	104	(61.2%)	
Live with adult(s) only	3753	738	(19.7%)	3016	(80.3%)	
Live with adult(s) and child(ren)	1460	379	(26.0%)	1081	(74.0%)	
**Age**						<0.001
18 to 29 years	858	287	(33.4%)	571	(66.6%)	
30 to 39 years	1879	500	(26.6%)	1379	(73.4%)	
40 to 49 years	1000	248	(24.8%)	752	(75.2%)	
50 to 59 years	1148	253	(22.0%)	895	(78.0%)	
60 to 69 years	1299	178	(13.7%)	1121	(86.3%)	
70+ years	837	59	(7.0%)	778	(93.0%)	
**Gender**						<0.001
Male	3484	666	(19.1%)	2818	(80.9%)	
Female	3482	840	(24.1%)	2642	(75.9%)	
Non-binary	55	19	(34.5%)	36	(65.5%)	
**Marital status**						<0.001
Married/living with partner	4398	880	(20.0%)	3518	(80.0%)	
Single/divorced/separated/widowed	2528	630	(24.9%)	1898	(75.1%)	
**Educational attainment**						0.276
High school or less	814	163	(20.0%)	651	(80.0%)	
Some post-secondary	1069	253	(23.7%)	816	(76.3%)	
College degree/diploma	1398	310	(22.2%)	1088	(77.8%)	
University degree/diploma	3679	795	(21.6%)	2884	(78.4%)	
**Household income (CAD)**						<0.001
Less than $40,000	853	251	(29.4%)	602	(70.6%)	
$40,000–79,999	1714	408	(23.8%)	1306	(76.2%)	
$80,000–119,999	1603	344	(21.5%)	1259	(78.5%)	
$120,000+	1706	322	(18.9%)	1384	(81.1%)	
Prefer not to answer	1145	200	(17.5%)	945	(82.5%)	
**Rurality**						0.006
Urban area	3268	760	(23.3%)	2508	(76.7%)	
Suburban area	2615	549	(21.0%)	2066	(79.0%)	
Rural area	1138	216	(19.0%)	922	(81.0%)	
**Survey wave**						0.003
1 (May 8 to May 12, 2020)	1005	256	(25.5%)	749	(74.5%)	
2 (May 28 to June 1, 2020)	1002	215	(21.5%)	787	(78.5%)	
3 (June 19 to 23, 2020)	1005	196	(19.5%)	809	(80.5%)	
4 (July 10 to 14, 2020)	1003	193	(19.2%)	810	(80.8%)	
5 (September 18 to 22, 2020)	1003	212	(21.1%)	791	(78.9%)	
6 (November 27 to December 1, 2020)	1003	244	(24.3%)	759	(75.7%)	
7 (March 19 to March 23, 2021)	1000	209	(20.9%)	791	(79.1%)	

Note: Totals do not match for all variables due to missing data.

Results from the unadjusted and multivariable logistic regression models are presented in Table 2. Household composition was significantly associated with anxiety symptoms both before and after adjusting for covariates. In the multivariable model, compared to those living alone, we observed significantly greater odds of anxiety symptoms among single parents/guardians (aOR = 2.00; 95% CI: 1.41–2.84), those living with adult(s) and child(ren) (aOR = 1.39; 95% CI: 1.10–1.76), and those living with adult(s) only (aOR = 1.22; 95% CI: 1.00–1.76). When comparing those living with adult(s) only to those living with adult(s) and child(ren), we did not find a significant odds ratio for anxiety symptoms (aOR = 1.14; 95% CI: 0.97–1.33; p = 0.108). However, when we collapsed “single parent/guardian” and “live with adult(s) and child(ren)” into one category–“live with child(ren)”–and re-ran the model we found that those living with child(ren) had significantly greater odds of anxiety symptoms compared to those living with adult(s) only (aOR = 1.19; 95% CI: 1.03–1.39; p = 0.020). No evidence of multicollinearity was found as all variance inflation factors were below 3.

**Table 2:** Unadjusted and multivariable logistic regression models for anxiety symptoms (GAD-7 10+), Canadian adults aged 18+ years during the COVID-19 pandemic.

	Unadjusted models Anxiety symptoms (GAD-7 10+)				Multivariable model Anxiety symptoms (GAD-7 10+)			
	n = 6,739				n = 6,739			
Variables	Crude ORs	95% CIs		Joint test Wald *χ*^2^ p-value	Adjusted ORs	95% CIs		Joint test Wald *χ*^2^ p-value
**Household composition**				**<0.001**				**<0.001**
Live alone	Ref	-	-		Ref	-	-	
Single parent/guardian	**2.57**	**1.84**	**3.60**		**2.00**	**1.41**	**2.84**	
Live with adult(s) only	0.97	0.83	1.13		**1.22**	**1.00**	**1.49**	
Live with adult(s) and child(ren)	**1.37**	**1.15**	**1.63**		**1.39**	**1.10**	**1.76**	
**Gender**				**<0.001**				**<0.001**
Male	Ref	-	-		Ref	-	-	
Female	**1.38**	**1.23**	**1.55**		**1.37**	**1.21**	**1.54**	
Non-binary	**2.23**	**1.16**	**4.28**		1.68	0.86	3.28	
**Age**				**<0.001**				**<0.001**
70+ years	Ref	-	-		Ref	-	-	
18 to 29 years	**6.99**	**5.12**	**9.55**		**6.27**	**4.53**	**8.67**	
30 to 39 years	**5.08**	**3.78**	**6.83**		**5.06**	**3.72**	**6.87**	
40 to 49 years	**4.56**	**3.34**	**6.23**		**4.42**	**3.19**	**6.11**	
50 to 59 years	**3.91**	**2.87**	**5.33**		**3.94**	**2.88**	**5.40**	
60 to 69 years	**2.18**	**1.58**	**3.00**		**2.21**	**1.60**	**3.05**	
**Marital status**				**<0.001**				0.204
Married/living with partner	Ref	-	-		Ref	-	-	
Single/divorced/separated/widowed	**1.33**	**1.18**	**1.49**		1.12	0.94	1.32	
**Educational attainment**				0.340				0.117
University degree / diploma	Ref	-	-		Ref	-	-	
High school or less	0.90	0.75	1.10		0.96	0.78	1.18	
Some post-secondary	1.11	0.94	1.31		**1.22**	**1.02**	**1.46**	
College degree/diploma	1.04	0.89	1.20		1.03	0.88	1.21	
**Household income (CAD)**				**<0.001**				**<0.001**
$120,000+	Ref	-	-		Ref	-	-	
Less than $40,000	**1.78**	**1.46**	**2.16**		**1.96**	**1.57**	**2.44**	
$40,000-$79,999	**1.32**	**1.12**	**1.56**		**1.43**	**1.19**	**1.71**	
$80,000-$119,999	1.16	0.97	1.38		1.19	0.99	1.42	
Prefer not to answer	0.97	0.79	1.18		1.08	0.88	1.34	
**Rurality**				**0.006**				0.060
Rural area	Ref	-	-		Ref	-	-	
Suburban area	1.15	0.96	1.38		1.09	0.90	1.32	
Urban area	**1.31**	**1.10**	**1.55**		**1.22**	**1.02**	**1.47**	
**Survey wave**				0.006				**0.003**
1 (May 8 to May 12, 2020)	Ref	-	-		Ref	-	-	
2 (May 28 to June 1, 2020)	0.83	0.67	1.03		0.83	0.66	1.03	
3 (June 19 to 23, 2020)	**0.73**	**0.59**	**0.91**		**0.72**	**0.58**	**0.90**	
4 (July 10 to 14, 2020)	**0.73**	**0.58**	**0.90**		**0.72**	**0.58**	**0.90**	
5 (September 18 to 22, 2020)	**0.78**	**0.63**	**0.97**		**0.79**	**0.63**	**0.98**	
6 (November 27 to December 1, 2020)	0.99	0.81	1.22		1.02	0.83	1.27	
7 (March 19 to March 23, 2021)	**0.79**	**0.64**	**0.97**		**0.79**	**0.64**	**0.99**	

Abbreviations: ORs = odds ratios; 95% CIs = 95% confidence intervals; Ref = reference category. Bolded figures indicate statistical significance (p<0.05).

As shown in Table 3, a modified least-squares model found that there was a statistically significant interaction between household composition and gender (*χ*^2^_[__2__]_ = 6.05; p = 0.0487), indicating that gender moderated the association between household composition and anxiety symptoms on the additive scale. Using the modified least-squares regression model from Table 3, prevalence differences were estimated for all levels of the interaction (see Table 4).

**Table 3:** Multivariable modified least-squares regression model with robust standard errors for anxiety symptoms (GAD-7 10+) including interaction terms (n = 6,698).

	Moderate to severe anxiety symptoms GAD-7 10+			
Variables	Adjusted PDs	95%CIs		Joint test Wald *χ*^2^ p-value
**Intercept**	-0.04	-0.10	0.01	0.133
**Household composition** (Ref = Live alone)				<0.001
Live with adult(s) only	0.04	0.00	0.08	
Live with child(ren)	0.10	0.05	0.15	
**Gender** (Ref = Male)				0.003
Female	0.06	0.02	0.10	
Household composition*Gender (Ref = Males living alone)				0.049
Females living with adult(s) only	0.00	-0.05	0.05	
Females living with child(ren)	-0.06	-0.12	0.00	
**Age** (Ref = 70+ years)				<0.001
18–29 years	0.24	0.20	0.28	
30–39 years	0.20	0.17	0.23	
40–49 years	0.17	0.13	0.20	
50–59 years	0.15	0.12	0.18	
60–69 years	0.07	0.04	0.09	
**Marital status** (Ref = Married/living with partner)				0.079
Single/divorced/separated/widowed	0.03	0.00	0.06	
**Education** (Ref = University degree / diploma)				0.176
High school or less	-0.01	-0.04	0.03	
Some post-secondary	0.03	0.00	0.06	
College degree / diploma	0.01	-0.02	0.03	
**Household income** (Ref = $120,000+)				<0.001
Less than $40,000	0.12	0.08	0.16	
$40,000-$79,999	0.06	0.03	0.09	
$80,000-$119,999	0.03	0.00	0.06	
Prefer not to answer	0.01	-0.02	0.04	
**Rurality** (Ref = Rural area)				0.042
Suburban area	0.02	-0.01	0.04	
Urban area	0.03	0.01	0.06	
**Survey wave** (Ref = 1 [May 8 to May 12, 2020])				0.005
2 (May 28 to June 1, 2020)	-0.03	-0.07	0.00	
3 (June 19 to 23, 2020)	-0.05	-0.09	-0.02	
4 (July 10 to 14, 2020)	-0.05	-0.09	-0.02	
5 (September 18 to 22, 2020)	-0.04	-0.08	0.00	
6 (November 27 to December 1, 2020)	0.00	-0.03	0.04	
7 (March 19 to March 23, 2021)	-0.04	-0.07	0.00	

Abbreviations: PDs = prevalence differences; 95% CIs = 95% confidence intervals; Ref = reference category.

Notes: Prevalence differences are on the probability scale. Non-binary gender group excluded due to small sample size.

**Table 4:** Prevalence differences for the association between household composition and anxiety symptoms (GAD-7 10+) modified by gender.

	Moderate to severe anxiety symptoms GAD-7 10+	
	Males	Females
Household composition	PD (95% CI)	PD (95% CI)
Live alone	Ref	0.06 (0.02, 0.10)
Live with adult(s) only	0.04 (0.00, 0.08)	0.11 (0.07, 0.15)
Live with child(ren)	0.10 (0.05, 0.15)	0.11 (0.06, 0.16)

Abbreviations: PD = prevalence difference; 95% CI = 95% confidence intervals; Ref = reference category.

Notes: Prevalence differences were estimated from the modified least-squares regression model presented in Table 3.

**Fig 1:**
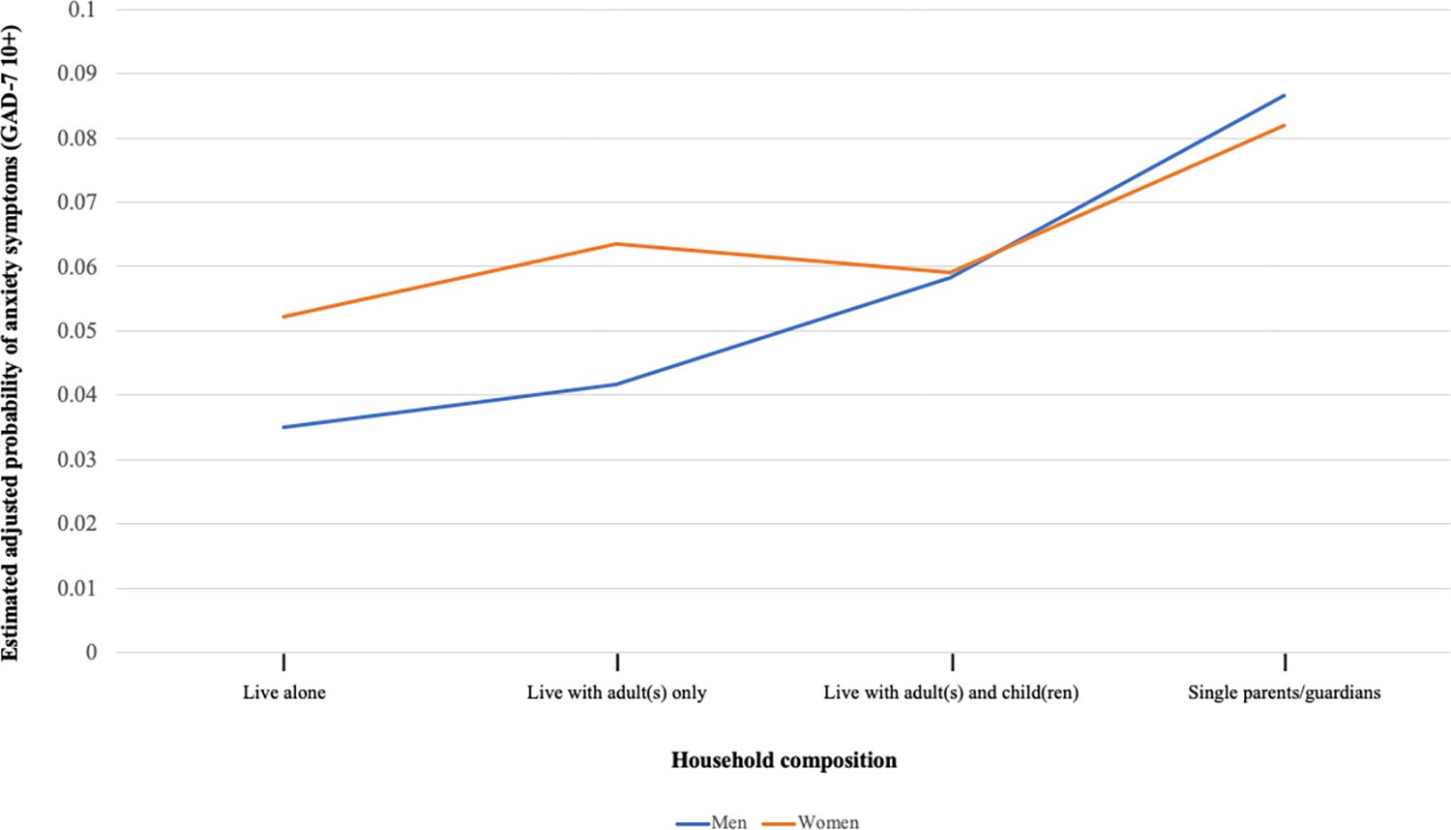
Illustration of the relationship between household composition and estimated adjusted probability of anxiety symptoms modified by gender. Model-estimated, covariate-adjusted probabilities of anxiety symptoms by gender and household composition. Probabilities were estimated from a logistic regression model (S1 Appendix), assuming all other covariates held at reference values (70+ years old, married/living with partner, university degree, $120k+ income, rural area, and survey wave 1).

Fig 1 illustrates the association between household composition and adjusted probability of anxiety symptoms stratified by gender, which were estimated from a multivariable logistic regression model. This graph shows that the association between living with child(ren)–both with and without other adult(s)–and anxiety symptoms, when compared to those living alone or with adult(s) only, was significantly greater among men compared to women.

## Discussion

We found that adults living with child(ren) under the age of 18 years–single parents/guardians in particular–had significantly greater odds of anxiety symptoms compared to those living alone. This finding is consistent with other population-based studies conducted during the pandemic [7, 27]. Adults living with children may be more vulnerable to the mental health effects of the pandemic for myriad reasons. With the intermittent closures of schools and daycare centres in response to the pandemic, parents have had to assume additional responsibilities for their children in the home [14, 28]. Single parents and guardians are especially affected because they lack the support of another caregiver in the home. During periods when schools and daycares were reopened, parents may have had anxiety about sending their children into environments that could potentially expose them to COVID-19. Parents may also be worried about financially providing for their children during a time of economic uncertainty, when unemployment reached its highest level (13.7%) since recordkeeping began in Canada in 1976 [29].

Gender moderated the association between household composition and anxiety symptoms such that the association between living with children and anxiety symptoms was stronger among men compared to women. It is possible that taking on additional caregiving responsibilities during the pandemic has been especially taxing on men who may be less familiar with having to balance work and parent duties and not as prepared to cope emotionally. Prior to the pandemic, research suggested that while mothers disproportionately experience parental burnout, it may be more detrimental to fathers’ wellbeing, contributing to escape ideation, suicidal ideation, and child neglect [30]. Moreover, recent research suggests that parental burnout may have increased more among fathers than among mothers during the pandemic [31], which may be contributing to greater increases in mental health and substance use problems among fathers [7, 14]. The current study further highlights the need to explore the experiences and mental health challenges of fathers, which has traditionally been a neglected group in mental health research [32].

Contrary to pre-pandemic research suggesting that living alone is positively associated with anxiety [13], we found that those living alone had significantly lower odds of anxiety symptoms compared to all other household compositions. Living under quarantine is associated with anxiety, anger, irritability, insomnia, low mood, and depression [33]. With people staying home together for extended periods of time, there is greater potential for conflict between family members and cohabitants, or even violence, which has reportedly increased in countries around the world during the COVID-19 pandemic [34]. Individuals living alone do not have the same potential for conflict in the home and presumably do not have childcare responsibilities in most cases, which may be protective against anxiety symptoms in the context of the pandemic. Individuals living alone also do not have to worry about themselves or others bringing COVID-19 into the home and infecting vulnerable loved ones, which is a burden associated with anxiety symptoms [35]. While individuals living alone appear to be experiencing anxiety symptoms less than other household compositions, we note that other research has linked living alone with depressive symptomatology during the pandemic, which may be related to social isolation and loneliness [27].

### Limitations and strengths

Several limitations should be acknowledged. Data were collected online and the response rate was modest, which could have introduced selection bias; however, it should be noted that 94% of Canadians have home Internet access [36], and that our sample was broadly representative of English-speaking Canadians in terms of age, gender, and region. The design of the study was cross-sectional, which makes it impossible to establish temporality between exposure and outcome. While we controlled for socioeconomic indicators, there could be residual confounding from overcrowded housing and the built environment, which are particularly important mental health factors when living under lockdown or stay-at-home orders [37]. Given that the survey we used did not specifically ask participants whether they were parents, our study cannot assume that adults living with children are parents or guardians, as they could be another relative (e.g., grandparent or sibling) or other person living in the same household; however, we note that the vast majority of adults living with children in Canada are parents [38]. We also note that the age cut-off for children was 18 years of age for the household composition measure, but some older adults live with children that are over 18 years of age, which could present a different set of challenges during the pandemic. Lastly, this study relied on self-report measures and used a screening tool for the anxiety symptoms outcome, which is not equivalent to a clinical diagnosis. However, the GAD-7 is a widely used screening tool in population surveys with established validity and strong psychometric properties.

Nevertheless, this study, which used a large nationally representative population-based sample in Canada, is one of the first to examine the relationship between household composition and anxiety symptoms during the COVID-19 pandemic. To our knowledge, it is the first to explore gender as an effect modifier for this relationship.

## Conclusion

Our findings suggest that additional supports are needed to address anxiety of adults living with children during infectious disease events such as the COVID-19 pandemic. Policymakers should aim to alleviate key parental stresses by providing parents with targeted financial support and virtual mental health services, by promoting community-based social support networks, and by ensuring that children can safely attend schools, daycares, and youth programs in person as the pandemic permits [7, 39–41]. Our findings also suggest that special consideration should be given to men living with children, including the development of evidence-based interventions anchored in acceptable lifestyle practices that account for men’s conceptualization, stigmatization, and concealment of mental health challenges and their gender roles and identities [42]. Research suggests that parent and child mental health are closely intertwined during the pandemic, with worse behavioural and mental health outcomes among children of distressed parents [41, 43, 44]. Distressed parents are less able to buffer against their child’s stresses, help them manage their emotions, and make sense of their experiences in the face of the pandemic [45]. Protecting the mental health of parents during this time is therefore important to prevent downstream increases in chronic mental health problems among adults and children alike.

## Supporting information

S1 Appendix (DOCX)
