# Supplementary material for: Household composition and anxiety symptoms during the COVID-19 pandemic: A population-based study
Source: PLoS One. 2022 Nov 3;17(11):e0277243. doi: 10.1371/journal.pone.0277243 (PMC9632761; doi:10.1371/journal.pone.0277243)
Supplement: S1 Appendix — (DOCX) [file pone.0277243.s001.docx]

**S1 Appendix: Multivariable logistic regression model used to estimate adjusted probabilities of anxiety symptoms (GAD-7 10+) for Figure 1 (n=6,698).**

|  | **Moderate to severe anxiety symptoms**  **GAD-7 10+** | | | |
| --- | --- | --- | --- | --- |
| **Variables** | **Coefficients**  **(log odds)** | **95%CI** | | **Joint test**  **p-value** |
| **Intercept** | -3.31 | -3.76 | -2.86 | <0.001 |
| **Gender** (Ref = Male) |  |  |  | 0.003 |
| Female | 0.42 | 0.14 | 0.69 |  |
| **Household composition** (Ref = Live alone) |  |  |  | 0.019 |
| Live with adult(s) only | 0.18 | -0.09 | 0.44 |  |
| Live with adult(s) and child(ren) | 0.53 | 0.23 | 0.83 |  |
| Single parent/guardian | 0.96 | 0.43 | 1.49 |  |
| **Household composition*gender** (Ref = Males living alone) |  |  |  | 0.013 |
| Females single parent/guardian | -0.48 | -1.18 | 0.22 |  |
| Females living with adult(s) only | 0.03 | -0.29 | 0.35 |  |
| Females living with adult(s) and child(ren) | -0.40 | -0.76 | -0.04 |  |
| **Age** (Ref = 70+ years) |  |  |  | <0.001 |
| 18-29 years | 1.86 | 1.53 | 2.19 |  |
| 30-39 years | 1.65 | 1.34 | 1.96 |  |
| 40-49 years | 1.49 | 1.16 | 1.82 |  |
| 50-59 years | 1.37 | 1.05 | 1.69 |  |
| 60-69 years | 0.80 | 0.48 | 1.13 |  |
| **Marital status** (Ref = Married/living with partner) |  |  |  | 0.178 |
| Single/divorced/separated/widowed | 0.12 | -0.05 | 0.29 |  |
| **Education** (Ref = University degree / diploma) |  |  |  | 0.152 |
| High school or less | -0.02 | -0.23 | 0.19 |  |
| Some post-secondary | 0.20 | 0.02 | 0.37 |  |
| College degree / diploma | 0.04 | -0.12 | 0.20 |  |
| **Household income** (Ref = $120,000+) |  |  |  | <0.001 |
| <$40,000 | 0.66 | 0.44 | 0.88 |  |
| $40,000-$79,999 | 0.35 | 0.17 | 0.53 |  |
| $80,000-$119,999 | 0.17 | -0.01 | 0.35 |  |
| Prefer not to answer | 0.08 | -0.14 | 0.29 |  |
| **Rurality** (Ref = Rural area) |  |  |  | 0.055 |
| Suburban area | 0.10 | -0.09 | 0.29 |  |
| Urban area | 0.21 | 0.02 | 0.40 |  |
| **Survey wave** (Ref = 1 [May 8 to May 12, 2020]) |  |  |  | 0.004 |
| 2 (May 28 to June 1, 2020) | -0.20 | -0.42 | 0.02 |  |
| 3 (June 19 to 23, 2020) | -0.32 | -0.55 | -0.10 |  |
| 4 (July 10 to 14, 2020) | -0.33 | -0.56 | -0.11 |  |
| 5 (September 18 to 22, 2020) | -0.24 | -0.47 | -0.02 |  |
| 6 (November 27 to December 1, 2020) | 0.02 | -0.20 | 0.23 |  |
| 7 (March 19 to March 23, 2021) | -0.23 | -0.45 | -0.01 |  |
| Notes: Ref = reference category; 95% CI = 95% confidence interval. Non-binary gender group excluded due to small sample size. Coefficients not reported as odds ratios due to difficulty in interpretability of effects involved in interactions. Interaction by gender is illustrated in Figure 1. | | | | |
